# Tunable ultraviolet and blue light generation from Nd:YAB random laser bolstered by second-order nonlinear processes

**DOI:** 10.1038/srep27107

**Published:** 2016-06-01

**Authors:** André L. Moura, Sandra J. M. Carreño, Pablo I. R. Pincheira, Zanine V. Fabris, Lauro J. Q. Maia, Anderson S. L. Gomes, Cid B. de Araújo

**Affiliations:** 1 Grupo de Física da Matéria Condensada, Núcleo de Ciências Exatas – NCEx, Campus Arapiraca, Universidade Federal de Alagoas, 57309-005, Arapiraca-AL, Brazil; 2 Departamento de Física, Universidade Federal de Pernambuco, 50670-901, Recife-PE, Brazil; 3 Grupo Física de Materiais, Instituto de Física, Universidade Federal de Goiás, 74001-970, Goiânia-GO, Brazil

## Abstract

Ultraviolet and blue light were obtained by nonlinear frequency conversion in a random laser (RL) based on Nd_0.10_Y_0.90_Al_3_(BO_3_)_4_ nanocrystalline powder. RL operation at 1062 nm, due to the ^4^F_3/2_ → ^4^I_11/2_ transition of neodymium ions (Nd^3+^), was achieved by exciting the Nd^3+^ with a tunable beam from 680 to 920 nm covering the ground state absorption transitions to the ^4^F_9/2_, (^4^F_7/2_,^4^S_3/2_), (^4^F_5/2_,^2^H_9/2_), and ^4^F_3/2_ states. Light from 340 to 460 nm was obtained via the second-harmonic generation of the excitation beam while tunable blue light, from 417 to 486 nm, was generated by self-sum-frequency mixing between the excitation beam and the RL emission.

Light generation and propagation in disordered media with gain can sustain laser oscillation due to stimulated emission and multiple scattering of light^1^,^2^,^3^. Owing to the disordered nature of the active medium, this type of laser is named random laser (RL). Since the beginning of the RL research large number of investigations concerned with the fundamentals^4^,^5^, demonstration of new effects^6^,^7^,^8^, novel operating architectures^9^,^10^,^11^,^12^,^13^,^14^, and several applications^15^,^16^,^17^ have been developed, as recently reviewed^18^.

Most of the RLs reported were operated by exciting the gain medium by a conventional laser that enables the observation of Stokes RL^18^. However, anti-Stokes RLs were also demonstrated by direct multi-photon excitation^9^,^19^ as well as by energy-transfer among neodymium ions (Nd^3+^) in a glassy powder^12^.

Another possibility to obtain anti-Stokes emission is by exploiting parametric nonlinear processes to convert the RL photons in more energetic ones. Second-harmonic generation in disordered systems provides wavelength conversions required for some applications^20^,^21^. In the special case of RLs, it was first demonstrated by Noginov *et al.*^22^ using a mixture of Nd_0.5_La_0.5_Al_3_(BO_3_)_4_ and frequency-doubling (2-methyl-4-nitroaniline) (MNA) particles. The Nd^3+^ of the Nd_0.5_La_0.5_Al_3_(BO_3_)_4_ particles were excited at 580 nm to generate the RL emission at 1063.0 nm and the interaction of the RL emission with the MNA particles produced the SH of the RL at 531.5 nm. Another approach to upconvert the RL emission was demonstrated using Nd_0.04_Y_0.96_Al_3_(BO_3_)_4_ nanocrystals thanks to their high second-order nonlinear coefficients^23^ and by the fact that laser emission at 1.06 μm is easily obtained in Nd^3+^-doped crystals. Multi-wavelength emission due to RL **self-frequency conversions** was demonstrated by exciting the nanocrystals at 806 nm, in resonance with the Nd^3+^ transition ^4^I_9/2_ → ^4^F_5/2_. The RL emission at 1062 nm (transition: ^4^F_3/2_→^4^I_11/2_) and frequency upconversion processes were possible due to **self**-second-harmonic generation (**self**-SHG) of the 1062 nm emission and **self**-sum-frequency generation (**self**-SFG) due to wave-mixing between the incident laser and the RL emission resulting in light generation at 459 nm^23^. In addition, the second-harmonic generation of the incident laser was observed at 403 nm. More recently^24^, the interplay between the RL process and the parametric conversions (**self-**SHG and **self**-SFG) in nanocrystalline powders of Nd_*x*_Y_1.00-*x*_Al_3_(BO_3_)_4_ for 0.05 ≤ *x *≤ 1.00 was investigated. The experiments in ref. 24 demonstrated that while the RL performance improved when *x* is increased, the intensity of the **self**-SFG signal decreased for *x *≥ 0.20 due to the crystalline phase change, from hexagonal to monoclinic, suffered by the nanocrystals. Tunable RL in the visible, near and mid-infrared have been demonstrated, as reviewed in ref. 18. An electrically pumped RL tunable from 352 to 377 nm has been demonstrated in MgZnO films^25^, whereas using a similar material, optically pumped tunable RL emission from 375 to 400 nm was also reported^26^.

In this paper, a tunable light source, emitting from 340 to 486 nm, based on the interplay between RL emission, **self**-SFG and second-harmonic generation is demonstrated for the first time to the best of our knowledge. The experiments were made with a Nd_0.10_Y_0.90_Al_3_(BO_3_)_4_ nanocrystalline powder and the motivation for the present work was the good RL performance and high second-order nonlinear response characterized in ref. 24 for this crystalline composition. The excitation laser wavelength, *λ*_*exc*_, was tuned from 680 to 920 nm, covering the Nd^3+^ absorption transitions from the ground state, ^4^I_9/2_, to the states ^4^F_9/2_, (^4^F_5/2_,^4^H_9/2_), (^4^F_7/2_,^4^S_3/2_) and ^4^F_3/2_. The second-harmonic (SH) generation of the excitation beam was from 340 to 460 nm while the blue light, from 417 to 486 nm, was obtained by **self**-SFG involving the excitation laser and the RL emission at 1062.0 nm. Self-second-harmonic generation (**self-**SHG) at 531.0 nm was also observed, as in ref. 23, but it is not discussed here because the wavelength is out of the ultraviolet-blue range of interest.

## Results

### Experimental details

Nd_0.10_Y_0.90_Al_3_(BO_3_)_4_ nanocrystals were synthesized by the polymeric precursor method. The diffraction patterns revealed a rhombohedral structure with R32 space group (hexagonal cell). The average particle’s size distribution, measured by TEM, has a broad dispersion, and presents large particles amount between 80 and 200 nm with peak around 120 nm. The details of the whole fabrication procedure as well as the structural and morphological nanocrystals characterization are described in detail in ref. 24.

The excitation of the nanocrystals was performed with an optical parametric oscillator (OPO) pumped by the SH of a Q-switched Nd:YAG laser (1064 nm; 7 ns, 5 Hz). The illuminated area in the sample surface was 1.2 mm^2^. The photoluminescence spectra were analyzed using a CCD coupled to a spectrometer, with resolution of ~0.1 nm. Decay-time measurements were performed by using a fast photodetector also coupled to the spectrometer. The excitation pulse energy (EPE) was controlled by a pair of polarizers, and the direction of the incident beam was 30° with respect to the normal of the sample. The light emitted by the powder was collected in the direction normal to the sample surface by a biconvex lens with focal length of 5 cm and diameter equal to 5 cm. The collimated light was focused on the spectrometer slit by a 20 cm focal length lens.

### Nd^3+^ random laser

**Figure 1:**
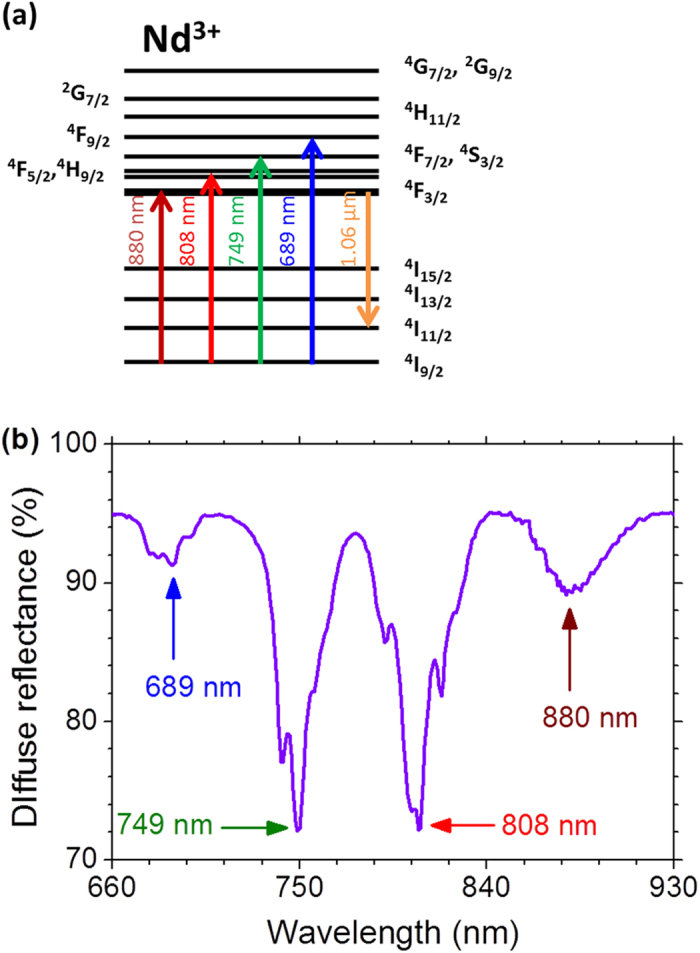
Summary of the Nd^3+^ random laser operation. (**a**) Simplified energy levels diagram of the Nd^3+^ ions. The upward arrows indicate the excitation wavelengths which correspond to the maximum of the absorption transitions and the downward arrow indicates the random laser transition at 1062 nm. (**b**) Diffuse reflectance spectrum of the Nd_0.10_Y_0.90_Al_3_(BO_3_)_4_ nanocrystals with the Nd^3+^ absorption transitions labeled by the peak wavelengths indicated in Fig. 1(a).

In order to generate RL emission due to the Nd^3+^ transition ^4^F_3/2_ → ^4^F_11/2_, the Nd^3+^ were excited to the states ^4^F_3/2_, (^4^F_5/2_, ^4^H_9/2_), (^4^F_7/2_, ^4^S_3/2_), and ^4^F_9/2_ that have energies corresponding to *λ*_*exc*_ = 880, 808, 749, and 689 nm, respectively, as indicated in Fig. 1(a). The corresponding absorption bands associated to those electronic transitions are shown in the diffuse reflectance spectrum presented in Fig. 1(b).

Characterization of the RL behavior was performed by exciting the powder and recording the emitted spectrum around 1060 nm for various EPE values. Increasing the EPE values the results showed bandwidth narrowing and an abrupt increase in the peak intensity related to the Nd^3+^ transition ^4^F_3/2_ → ^4^F_11/2_ at 1062.0 nm, which is the maximum of the gain curve, when crossing the EPE threshold (EPE_th_) as described in details in refs 23 and 24. The transition from luminescence to RL was corroborated by the decay-time measurements of the luminescence from the ^4^F_3/2_ energy level: for EPE < EPE_th_ a decay of ≈45 μs was recorded, while for EPE > EPE_th_ the decay-time shortened to about 7 ns.

**Figure 2:**
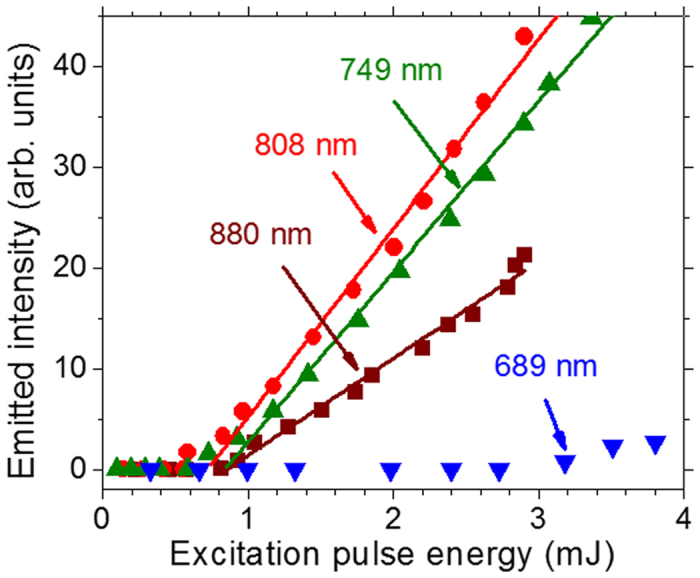
Random laser slope efficiency. Intensity dependence of the emission at 1062.0 nm with the excitation pulse energy for the four excitation wavelengths, in resonance with Nd^3+^ transitions starting from the ground state, indicated in Fig. 1(a). Error bars are comparable or smaller than the symbols representing the measured values. The illuminated area of the sample and the excitation pulse duration were 1.2 mm^2^ and 7 ns, respectively.

Figure 2 displays the RL intensity dependence versus the EPE from where one can see that the most efficient wavelength for RL excitation is *λ*_*exc*_ = 808 nm that corresponds to the lower EPE_th_ and the larger slope efficiency.

### Ultraviolet and blue light generation

Additionally to the RL emission, the excitation around the four absorption bands shown in Fig. 1(b), enabled the observation of SH of the excitation beam, **self-**SHG of the RL, and **self-**SFG by the wave-mixing between the RL and the excitation beam. These generation processes are efficient because of the high second-order nonlinear coefficients of the Nd_0.10_Y_0.90_Al_3_(BO_3_)_4_ nanocrystals^24^.

**Figure 3:**
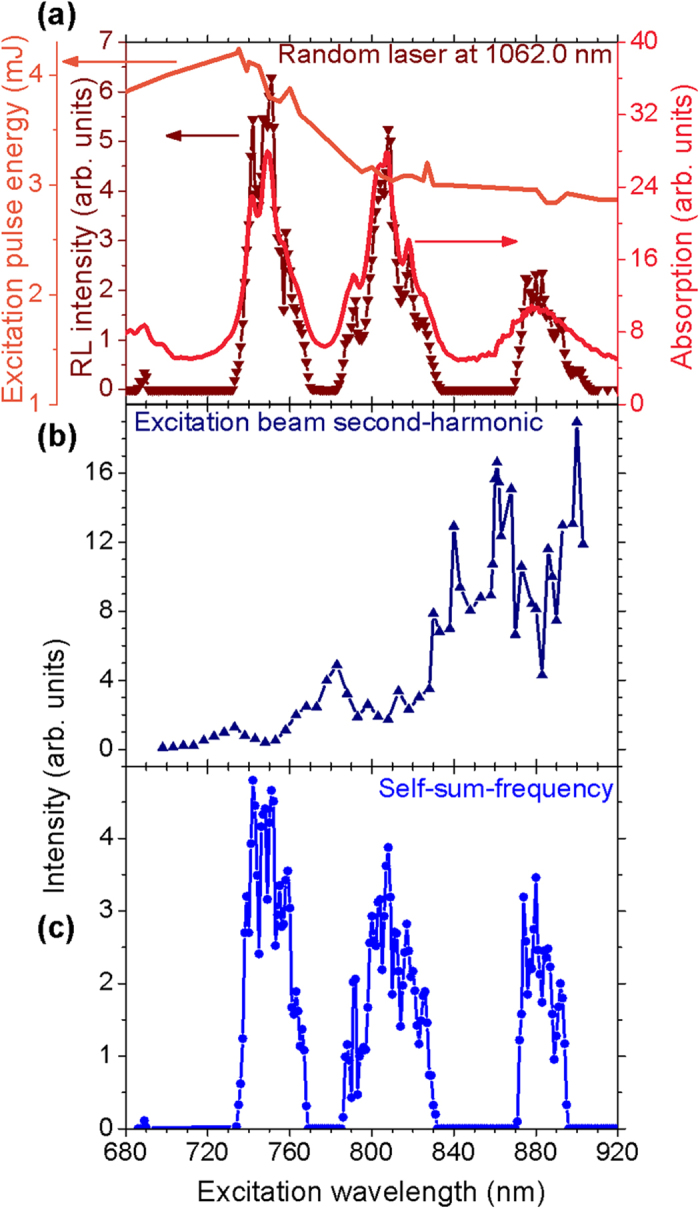
Emitted intensities versus the excitation wavelength. (**a**) Excitation spectrum of the random laser (RL) at 1062 nm. The powder absorption and the excitation pulse energy spectra are also presented. Excitation spectra of the second-harmonic of the excitation of the RL (**b**) and of the self-sum-frequency generation (**c**) due to the wave-mixing between the RL and the excitation beam. Error bars are not shown in the figure because they are small or does not provide any relevant information for interpretation of the data. The illuminated area of the sample and the pulse duration were 1.2 mm^2^ and 7 ns, respectively.

Figure 3(a) shows the 1062.0 nm RL excitation spectrum obtained with the maximum EPE available from the OPO used. For excitation wavelengths between two successive Nd^3+^ absorption bands no RL emission is observed. Also, the absorption spectrum (obtained from the diffuse reflectance spectrum) is shown in Fig. 3(a) as a function of the excitation wavelength. The RL emission mimics the absorption spectrum and the relative intensities among the three peaks in the emission spectrum are influenced by the different absorption coefficients, light scattering cross-section at each *λ*_*exc*_ and also due a small change of the OPO output energy while scanning the spectral range, as showed in Fig. 3(a). Figure 3(b) shows the behavior of the excitation beam SH between 340 and 460 nm for *λ*_*exc*_ varying from 680 to 920 nm. The intensity growth with *λ*_*exc*_ was not corrected with respect to the spectral response of the CCD used for acquiring the data. No data are shown for excitation around 680 nm due to the lower CCD sensitivity. The valleys centered at 749, 808, and 880 nm are due to the large efficiency conversion of the excitation beam to RL emission. Figure 3(c) presents the excitation spectrum for the **self-**SFG, which depends on both the excitation beam and the RL emission. For excitation wavelengths which do not generate RL emission, the **self-**SFG is not observed.

We remark that the spectrum reported in Fig. 3(c) illustrates the first operation of a tunable anti-Stokes source of radiation based on a RL. The wavelengths emitted in the 417 to 486 nm range (*λ*_*SSFG*_) are due to the **self-**SFG between the RL and *λ*_*exc*_ that was tuned from 680 to 920 nm.

**Figure 4:**
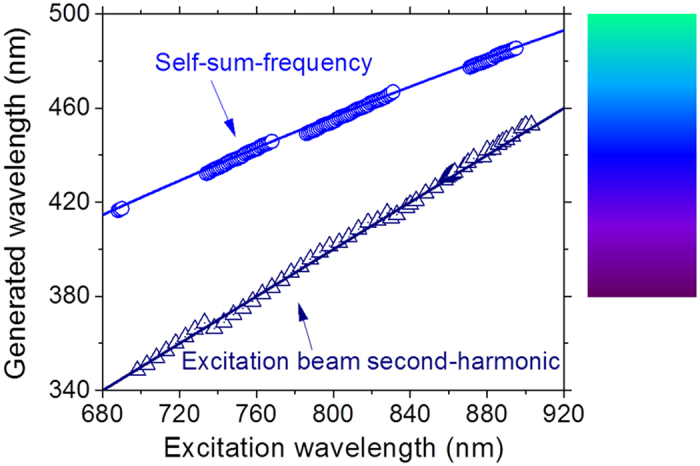
Ultraviolet and blue light wavelengths as a function of the excitation wavelength. The solid lines connecting the data represent the second-harmonic and the self-sum-frequency generation expected considering the incident and the RL wavelengths. The colored panel shows the color of the generated wavelengths. Error bars are smaller than the symbols representing the measured values.

The results of Fig. 3 are summarized in Fig. 4 which also displays the theoretical wavelengths expected for both processes, that are given by 
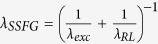
, with *λ*_*RL*_ = 1062.0 nm, and 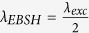. The lack of experimental **self-**SFG data in two spectral windows is because of the absence of RL emission when *λ*_*exc*_ assumes values between two successive Nd^3+^ absorption bands.

The reproducibility of the presented results is assured by the non-degradation of the Nd:YAB nanoparticles under laser exposure.

## Discussion

Tunable RL sources have been described with somewhat limited spectral range in the visible region, as reviewed in ref. 18. In the UV region, tunability range up to 25 nm have been reported^25^,^26^. By exploiting the interplay between RL emission and second order wave mixing processes, we were able to demonstrate a tunability range of 120 nm due to a combination of SH and **self-**SFG in the Nd^3+^ powder. The tunable light source reported can find biomedical applications, such as in photodynamic therapy where the main drawback is the strong blue light absorption of the human tissues. This problem can be avoided by infiltrating the nanocrystals into the tissues that can be excited through the biological near-infrared window generating ultraviolet-blue light by **self-**SFG deep inside the tissue. Another potential application of the results herein reported is the use of Nd_0.10_Y_0.90_Al_3_(BO_3_)_4_ nanocrystals as multifunctional bio-markers nanoprobes, for example, to investigate cortical neurons^27^,^28^ since the upconverted emissions present narrow linewidths.

Co-doping the nanocrystals with other trivalent rare-earth ions would fill the spectral gaps shown in Fig. 4 and provide a continuous spectrum in the ultraviolet-blue region via wave-mixing of the RL emission and the excitation laser beam. The availability of a tunable ultraviolet-blue source of low coherence as the one reported here may be very useful for imaging applications^17^.

As above-mentioned, the Nd_0.10_Y_0.90_Al_3_(BO_3_)_4_ nanoparticles were used because it presented the best RL and **self**-frequency conversions performances among the Nd_*x*_Y_1.00-*x*_Al_3_(BO_3_)_4_ (0.05 ≤ *x *≤ 1.00) particles. In a forthcoming publication, the system Nd_0.10_Y_0.90_Al_3_(BO_3_)_4_ will be optimized with respect to the particles’ size focusing at the performances of the RL and the nonlinear frequency conversions. This work will require a detailed study of light scattering and phase-matching conditions for the nonlinear conversions due to the different behavior of the RL^29^ and wave-mixing processes with the particles’ size^20^,^30^.

In summary, due to the high laser efficiency of the Nd^3+^ transition ^4^F_3/2_ → ^4^F_11/2_ and the large second-order nonlinear coefficients of the Nd_0.10_Y_0.90_Al_3_(BO_3_)_4_ nanocrystals, tunable anti-Stokes ultraviolet-blue light generation was demonstrated for the first time using a random laser. The blue emission, from 417 to 486 nm, was achieved due to the self-sum-frequency generation (**self-**SFG) between the excitation beam, tuned from 680 to 920 nm, and the RL emission at 1062.0 nm. Additionally, the second-harmonic of the excitation beam was also observed from 340 to 460 nm.

## Additional Information

**How to cite this article**: Moura, A.L. *et al.* Tunable ultraviolet and blue light generation from Nd:YAB random laser bolstered by second-order nonlinear processes. *Sci. Rep.* **6**, 27107; doi: 10.1038/srep27107 (2016).
